# Cognitive Function Declines Following Orthostatic Stress in Adults With Myalgic Encephalomyelitis/Chronic Fatigue Syndrome (ME/CFS)

**DOI:** 10.3389/fnins.2020.00688

**Published:** 2020-06-26

**Authors:** C. (Linda) M. C. van Campen, Peter C. Rowe, Freek W. A. Verheugt, Frans C. Visser

**Affiliations:** ^1^Stichting CardioZorg, Hoofddorp, Netherlands; ^2^Department of Pediatrics, Johns Hopkins University School of Medicine, Baltimore, MD, United States; ^3^Onze Lieve Vrouwe Gasthuis, Amsterdam, Netherlands

**Keywords:** N-back cognitive test, orthostatic intolerance, tilt table test, myalgic encephalomyelitis, chronic fatigue syndrome

## Abstract

**Introduction:**

Orthostatic intolerance (OI) is common among individuals with myalgic encephalomyelitis/chronic fatigue syndrome (ME/CFS). Cognitive dysfunction has been demonstrated during head-up tilt testing (HUT) in those with ME/CFS: worse scores on cognitive tests occur with increasing tilt angles and increasing complexity of the cognitive challenge. The aim of our study was to determine whether cognitive impairment persists after completion of HUT.

**Methods and Results:**

Eligible participants were consecutive individuals satisfying criteria for ME/CFS who underwent HUT because of OI. The 2- and 3-back tests were performed before the start of HUT and within 5 min after completion of HUT. We measured the percentage of correct responses and raw reaction times before and after HUT for both the 2- and 3-back tests. We studied 128 ME/CFS patients who underwent HUT and had a complete set of N-back data before and after HUT. Compared to pre-tilt responses, the percentage of correct responses on the 2-back test decreased post-HUT from 77(18) to 62(21) and of the 3-back test from 57(17) to 41(17) (both *p* < 0.0001). The raw reaction time of the 2-back test increased post-HUT from 783(190) to 941(234) m/s and of the 3-back test from 950(170) to 1102(176) (both *p* < 0.0001). There was no difference in the N-back test data for subgroups dichotomized based on disease severity, the presence of co-morbid fibromyalgia, or the presence of postural orthostatic tachycardia syndrome.

**Conclusion:**

As measured by the N-back test, working memory remains impaired in adults with ME/CFS following a 30-min head-up tilt test.

## Introduction

In 1969, myalgic encephalomyelitis (ME/CFS) was introduced into the eighth edition of the international classification of diseases of the WHO (ICD-8: code 323) and had been classified as a disease of the central nervous system (Briggs, 1970). Chronic fatigue syndrome (CFS) was added to the ICD-9. Because of a substantial overlap of clinical features and the absence of a diagnostic biomarker that discriminates between these two, many refer to this disease as ME/CFS. Studies in the 1990s highlighted the association between ME/CFS and various forms of orthostatic intolerance (OI), such as orthostatic hypotension (OH) and postural orthostatic tachycardia syndrome (POTS) (Bou-Holaigah et al., 1995; De Lorenzo et al., 1997; Freeman and Komaroff, 1997; Stewart et al., 1999; Streeten and Bell, 1999). Although neglected in the Fukuda criteria for CFS (Fukuda et al., 1994) OI is one of several qualifying features in the international consensus criteria (ICC) for ME (Carruthers et al., 2011) and a cardinal feature in the United States (Institute of Medicine [IOM], 2015).

Impairments in cognitive functioning are among the most frequently reported symptoms of ME/CFS. Patients describe these cognitive symptoms as equally debilitating compared to the physical symptoms that accompany this disease. During a survey of ME/CFS patients, the descriptions of the memory and concentration problems were variously described as: brain fog, confusion, disorientation, hard to concentrate, can’t focus, inability to process information, inability to multi-task, and short-term memory loss. In more severe cases, patients have difficulty completing tasks that require sustained attention and report problems performing even relatively simple activities such as watching television (FDA, 2013). Patients report slowed information processing, poor memory function compared to the time before the disease started, and overall mental fatigue or slowed thinking (Larun and Malterud, 2007; Constant et al., 2011). One of the best studied aspects of ME/CFS is cognition. In a meta-analysis of 50 studies using a total of 80 cognitive tests with 79 different scores, of 8 cognitive domains described, reaction time and attention were the only two domains with a moderate to large, significant difference between ME/CFS patients and healthy controls (Cockshell and Mathias, 2010).

One of the tests used for investigating working memory processes is the N-back test. The N-back test is a continuous performance measure where stimulus sequences of visual, auditory, or olfactory stimuli are presented and the subject is required to indicate whether the actual stimulus matches the one presented “n” trials previously: for a visual 2-back only one other picture between the two same stimuli is required, and for a visual 3-back two other pictures between the two same stimuli are required (Owen et al., 2005). Slowed information processing is the most commonly reported objective neurocognitive symptom in ME/CFS patients (Institute of Medicine [IOM], 2015; Mahurin et al., 2004; Claypoole et al., 2007; Togo et al., 2015). As the N-back test is dependent on processing information speed in the working memory, the test has been used to measure cognitive function in ME/CFS patient groups (Owen et al., 2005; Cockshell and Mathias, 2010; Stewart et al., 2012; Medow et al., 2014).

As one of the symptoms in OI syndromes is impaired concentration due to cerebral underperfusion (Low et al., 2009), we hypothesized that cognitive deterioration in ME/CFS patients would be present after orthostatic stress induced by the head-up tilt test (HUT). For this purpose the raw reaction times and percentage correct answers of the 2- and 3-back test were analyzed pre-and post-HUT in ME/CFS patients. Moreover, cognitive dysfunction is part of the post-exertional malaise and the onset of the post-exertional malaise is variably reported in literature, from immediately after the stressor up until days later (Sorensen et al., 2003; Yoshiuchi et al., 2007; van Oosterwijck et al., 2010; White et al., 2010). Therefore, we assessed cognitive decline immediately after HUT.

## Materials and Methods

### Eligible Participants

Individuals were eligible for this study if they were evaluated between November 2015 and June 2018, met the criteria for ME/CFS, and underwent a HUT to evaluate a clinical suspicion of OI. OI was defined as described in the IOM report: “Orthostatic intolerance is defined as a clinical condition in which symptoms worsen upon assuming and maintaining upright posture and are ameliorated (although not necessarily abolished) by recumbency” [(IOM) 2015]. Symptoms of orthostatic intolerance sought in the history of patients “are those caused primarily by (1) cerebral underperfusion (such as light- headedness, near-syncope or syncope, impaired concentration, headaches, and dimming or blurring of vision), or (2) sympathetic nervous system activation (such as forceful beating of the heart, palpitations, tremulousness, and chest pain. Other common signs and symptoms of orthostatic intolerance are fatigue, a feeling of weakness, intolerance of low-impact exercise, nausea, abdominal pain, facial pallor, nervousness, and shortness of breath.” We included all those in whom a complete set of N-back tests were available. ME/CFS was considered present if participants met both the 1994 International Chronic Fatigue Syndrome Study Group criteria for CFS (Fukuda et al., 1994) and the 2011 international consensus definition of ME (Carruthers et al., 2011), taking the exclusion criteria into account.

The study was carried out in accordance with the Declaration of Helsinki. The use of clinical data for descriptive studies was approved by the ethics committee of the Slotervaart Hospital, the Netherlands (P1450). All patients gave informed consent to analyze their data.

### Head-Up Tilt Table Test

The HUT was performed as described previously (van Campen et al., 2018). Briefly, testing was conducted at least 3 h after a light meal. Participants were encouraged to ingest an ample amount of fluid on the day of the procedure, but did not drink fluids in the 2 h before the test. Participants were studied in a climate-controlled room where the temperatures ranged from 22–24°C. Individuals were studied in the supine position for 15 min, and for 30 min in the upright position (70-degrees). The test was ended after 30 min, at the request of the patient, or if the individual developed syncope or pre-syncope.

Heart rate (HR), systolic and diastolic blood pressures (SBP and DBP) were continuously recorded by finger plethysmography using the Nexfin device (BMeye, Amsterdam, Netherlands) (Eeftinck et al., 2009; Martina et al., 2012). An independent radio-controlled clock was used to mark the starting time of HR and BP recordings as well as the time of the start of tilting. HR and BP data were extracted from the Nexfin device and imported into an Excel spreadsheet. Supine HR and BP data were calculated from the last minute data before tilting. Upright HR and BP data were calculated from the last minute data of the upright position. HR and BP responses during the HUT were classified according to consensus guidelines, like orthostatic hypotension (a decrease of over 20 mmHg in systolic blood pressure and over 30 mmHg in case of a systolic blood pressure over 140 mmHg, or a decrease of 10 mmHg in diastolic blood pressure) and postural orthostatic tachycardia syndrome (a sustained increase of at least 30 bpm within 10 min, without a significant decrease in BP) (Freeman et al., 2011; Sheldon et al., 2015). Nasal prongs were placed to measure expired carbon dioxide (CO_2_) concentrations. For the tilt testing component, individuals being treated with medication that could alter HR or BP (beta-adrenergic antagonists, midodrine, fludrocortisone, desmopressin, pyridostigmine bromide, anti-hypertensive medications, or ivabradine) were excluded from this analysis. Individuals being treated with selective serotonin reuptake inhibitors or serotonin norepinephrine reuptake inhibitors continued to take these medications.

### N-Back Cognitive Test

We used a visual N-back test available online: http://cognitivefun.net/test/4. The visual N-back test is composed of stimuli of 10 different simple colored cartoons randomly shown on the screen. The stimulus interval was approximately 1900 m/s and each stimulus was shown for approximately 1500 m/s. All participating ME/CFS patients were required to log in at the website and perform several training sessions of the visual 2- and 3-back tests before they underwent HUT. Patients were excluded when they performed each test less than 10 times because of the learning curve of the test. All tests before and after HUT were executed on the same computer system with the same right-handed mouse pointer. Fifteen minutes before the start of the HUT, the patients were asked to perform the visual 2- and 3-back test. The software shows the correct or incorrect answers on the screen, just to the right of the presented cartoon. To avoid distracting the participants this part of the screen was blinded during data acquisition. Within 5 min after finalization of the HUT, the visual 2- and 3-back tests were repeated. From the tests the percentage of correct responses and the raw reaction time in m/s were noted.

### Statistical Analysis

Data were analyzed using the statistical package of Graphpad Prism version 8.2.4 (Graphpad software, La Jolla, CA, United States). All continuous data were tested for normal distribution using the Kolmogorov–Smirnov test, and presented as means (SD) or as median with the IQR where appropriate. Nominal data were compared using the Chi-square test (in a 3 × 2 table). For continuous data groups were compared using the paired or unpaired *t*-test where appropriate. Within group comparison was done by the two-way analysis of variance (ANOVA). Where significant, results were then explored further using the *post hoc* Holm–Sidak test. A *p*-value of <0.05 was considered to be statistically significant.

## Results

We evaluated 385 individuals with ME/CFS and a clinical suspicion of OI at the Stichting CardioZorg during the study period. We excluded those with another type of orthostatic stress testing (seated test or active standing test: *n* = 16), those who had not completed training on the test due to the absence of a laptop or computer at home (*n* = 118), those who were left-handed (*n* = 9), and those who had not trained sufficiently as per protocol (*n* = 111). Three others were excluded because of pre- syncope and not being able to perform the N-back tests within 5 min after HUT (*n* = 3). None of the patients used HR or BP lowering drugs before the HUT. This left 128 participants to be analyzed. Demographic data of the patients not analyzed were comparable to the demographic data of the patients included in the analysis (data not shown).

Table 1 shows the demographic characteristics of the study population. During history taking at the first visit, 84% percent reported memory and concentration problems (108/128). Based on the history taking at the first visit, ME/CFS severity was graded as mild in 49 (38%), moderate in 55 (43%), and severe in 24 (19%) according to the ME criteria (Carruthers et al., 2011).

**TABLE 1:** Demographic data and hemodynamic HUT results of the study population.

**Demographic data**	
Number of patients	128
Females	116/128 (91%)
Height in cm	172 (8)
Weight in kg	75 (17)
Age in years	39 (11)
Median duration of ME/CFS (IQR) in years	9 (5–16)
Disease severity: mild/moderate/severe*	49/55/24 (38%/43%/19%)
Fibromyalgia present	65/128 (51%)
Self-reported cognitive problems	108/128 (84%)
SSRI use	34/128 (27%)
**Hemodynamic responses during HUT**	
Normal heart rate/blood pressure response	59/128 (46%)
Postural orthostatic tachycardia syndrome (POTS)	49/128 (38%)
Orthostatic hypotension (OH)	20/128 (16%)

Data represent mean (SD). HUT: head-up tilt test; SSRI: selective serotonin reuptake inhibitors. *Disease severity grading according to classification of ICC (Carruthers et al., 2011).

Table 2 shows the hemodynamic results of the HUT in ME/CFS patients with normal heart rate/blood pressure response (norm HR/BP) (*n* = 59), in ME/CFS patients with postural orthostatic tachycardia syndrome (POTS) (*n* = 49) and ME/CFS patients with orthostatic hypotension (OH) (*n* = 20). By definition, the HR increase in the POTS group and the BP decline in the OH group are significantly different from the two other groups. The 2-way ANOVA showed a significant interaction effect between the three predefined hemodynamic profiles and the results of heart rate, systolic blood pressure, diastolic blood pressure and end-tidal CO_2_ (*p*-value varying between 0.0038 and <0.0001). *Post hoc* analysis results are presented in the table.

**TABLE 2:** Hemodynamic responses during HUT of the study population.

	**Group 1 NormHR/BP**	**Group 2 POTS**	**Group 3 OH**	**2-way ANOVA and *post hoc* Holm-Sidak test**
Number of patients	59	49	20	
Male/female	8/51	3/46	1/19	Chi square 0.32 (3 × 2 table)
HR supine (bpm)	74 (11)	80 (15)	68 (9)	*F* (2, 250) = 11.10; *p* < 0.0001. *Post hoc* tests: pre-HUT 1 vs. 2 *p* = 0.052 1 vs. 3 *p* = 0.096 and 2 vs. 3 *p* = 0.0038 and post-HUT 1 vs. 2 *p* < 0.0001; 1 vs. 3 *p* = 0.58 and 2 vs. 3 *p* < 0.0001
End of tilt HR (bpm)	91 (11)	118 (18)	89 (18)	
SBP supine (mmHg)	137 (17)	132 (12)	142 (11)	*F* (2, 250) = 13.37; *p* < 0.0001. *Post hoc* tests: pre-HUT 1 vs. 2 *p* = 0.18 1 vs. 3 *p* = 0.21 and 2 vs. 3 *p* = 0.046 and post-HUT 1 vs. 2 *p* = 0.008; 1 vs. 3 *p* < 0.0001 and 2 vs. 3 *p* = 0.0076
End of tilt SBP (mmHg)	131 (18)	123 (15)	111 (15)	
DBP supine (mmHg)	80 (8)	79 (7)	78 (6)	*F* (2, 250) = 9.008; *p* = 0.0002. *Post hoc* tests: pre-HUT 1 vs. 2 *p* = 0.79 1 vs. 3 *p* = 0.74 and 2 vs. 3 *p* = 0.79 and post-HUT 1 vs. 2 *p* = 1.0; 1 vs. 3 *p* < 0.0001 and 2 vs. 3 *p* < 0.0001
End of tilt DBP (mmHg)	85 (9)	85 (9)	73 (11)	
EtCO_2_ supine (mmHg)	37 (3)	36 (3)	37 (3)	*F* (2, 250) = 5.69; *p* = 0.0038. *Post hoc* tests: pre-HUT 1 vs. 2 *p* = 0.56 1 vs. 3 *p* = 1.0 and 2 vs. 3 *p* = 0.63 and post-HUT 1 vs. 2 *p* < 0.0001; 1 vs. 3 *p* = 0.078 and 2 vs. 3 *p* = 0.0.0013
End of tilt EtCO_2_ (mmHg)	32 (5)	26 (6)	30 (5)	

*Data represent mean (SD). All upright data are end of tilt test data. DBP: diastolic blood pressure; EtCO_2_: end-tidal CO_2_; HR: heart rate; NormHR/BP: normal heart rate/blood pressure response; OH: orthostatic hypotension; POTS: postural orthostatic tachycardia syndrome; SBP: systolic blood pressure.*

Table 3 shows the N back results between the groups in the ME/CFS patients with a normal heart rate and blood pressure response, the ME/CFS patients with POTS and the ME/CFS patients with orthostatic hypotension. All N-back results (percent correct responses and raw reaction times for both 2-back and 3-back) were compared pre- and post-HUT. All parameters changed highly significantly different (*p* all <0.0001). The 2-way ANOVA showed no significant within group differences and no significant interaction effect between the three hemodynamic profiles and the pre- and post-HUT N-back results for both 2-back and 3-back.

**TABLE 3:** N-back results pre- and post-HUT in ME/CFS patients with norm HR/BP, POTS and orthostatic hypotension.

**N-back results**	**Group 1 Norm HR/BP**	**Group 2 POTS**	**Group 3 OH**	**2-way ANOVA and *post hoc* Holm–Sidak test**
2 Back test	*n* = 59	*n* = 49	*n* = 20	
% Correct response pre-HUT	80 (15)	76 (19)	73 (19)	*F* (2, 250) = 0.18; *i* = 0.83.
% Correct response post-HUT	67 (20)	59 (23)	58 (20)	
Raw reaction time pre-HUT	774 (192)	793 (191)	790 (190)	*F* (2, 250) = 0.038; *p* = 0.96.
Raw reaction time post-HUT	941 (243)	939 (223)	945 (247)	
3 Back test				
% Correct response pre-HUT	60 (17)	57 (18)	52 (13)	*F* (2, 250) = 0.081; *p* = 0.92.
% Correct response post-HUT	44 (17)	41 (18)	34 (12)	
Raw reaction time pre-HUT	945 (171)	939 (166)	966 (180)	*F* (2, 250) = 0.14; *p* = 0.87.
Raw reaction time post-HUT	1100 (175)	1085 (190)	1142 (143)	

*Data represent mean (SD). Raw reaction time is given in m/s. HUT: head-up tilt test; Norm HR/BP: normal heart rate and blood pressure response of the head-up tilt test; POTS: postural orthostatic tachycardia syndrome.*

**FIGURE 1:**
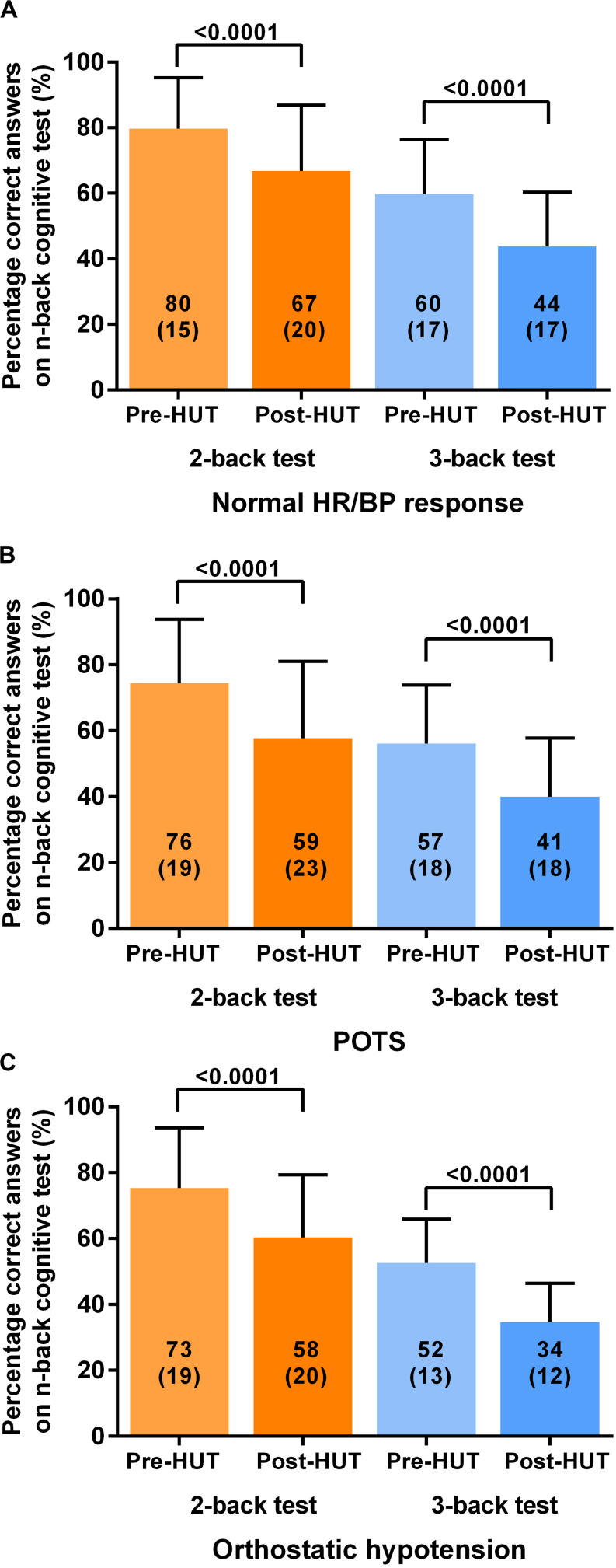
Shows the percentage of correct responses on 2-back and 3-back test before and after head-up-tilt testing for the three HUT results: normal heart rate and blood pressure **(A)**, POTS **(B)** and orthostatic hypotension **(C)**. HUT: head-up tilt test; Norm HR/BP response: normal heart rate and blood pressure response.

**FIGURE 2:**
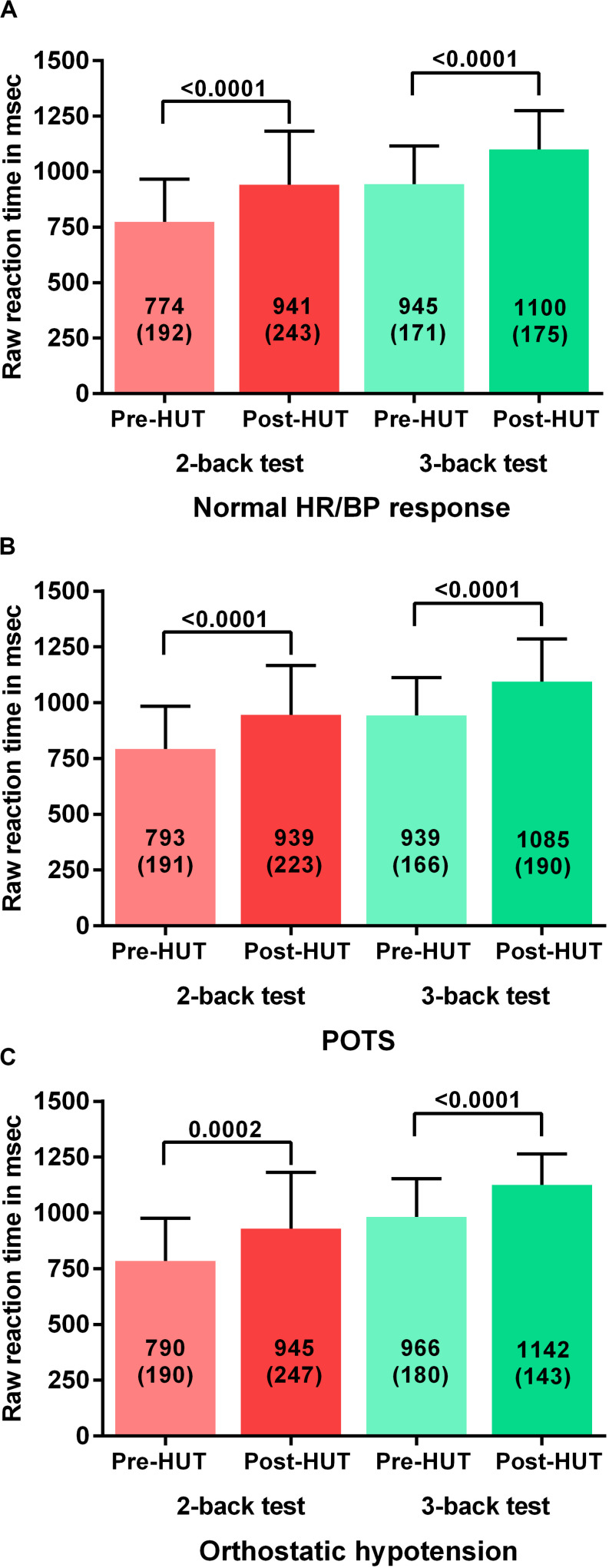
Shows the raw reaction time on 2-back an 3-back test before and after head-up-tilt testing for the three HUT results: normal heart rate and blood pressure **(A)**, POTS **(B)**, and orthostatic hypotension **(C)**. HUT: head-up tilt test; Norm HR/BP response: normal heart rate and blood pressure response.

For all patients the percentage of correct responses on 2- and 3 back test before and after HUT showed a significant reduction: in the 2-back from 77(18) to 62(21) and in the 3-back from 57(17) to 41(17) (both *p* < 0.0001). Figure 1 shows the percentage of correct responses of 2- and 3-back tests before and after HUT in the three different hemodynamic groups: normal heart rate and blood pressure response (Figure 1A), POTS (Figure 1B) and orthostatic hypotension (Figure 1C). In all three groups a significant reduction of the percent correct responses was found (all *p* < 0.0001). For all patients the raw reaction times on 2- and 3-back tests before and after HUT showed a significant increase: the 2-back from 783(190) to 941(234) m/s and the 3-back from 950(170) to 1102(176) m/s (both *p*: < 0.0001). Figure 2 shows the raw reaction time of 2- and 3-back tests before and after HUT in the three different hemodynamic groups: normal heart rate and blood pressure response (Figure 2A), POTS (Figure 2B) and orthostatic hypotension (Figure 2C). In all three groups a significant increase in raw reaction time was found (*p* ranging between 0.0002 and <0.0001).

**FIGURE 3:**
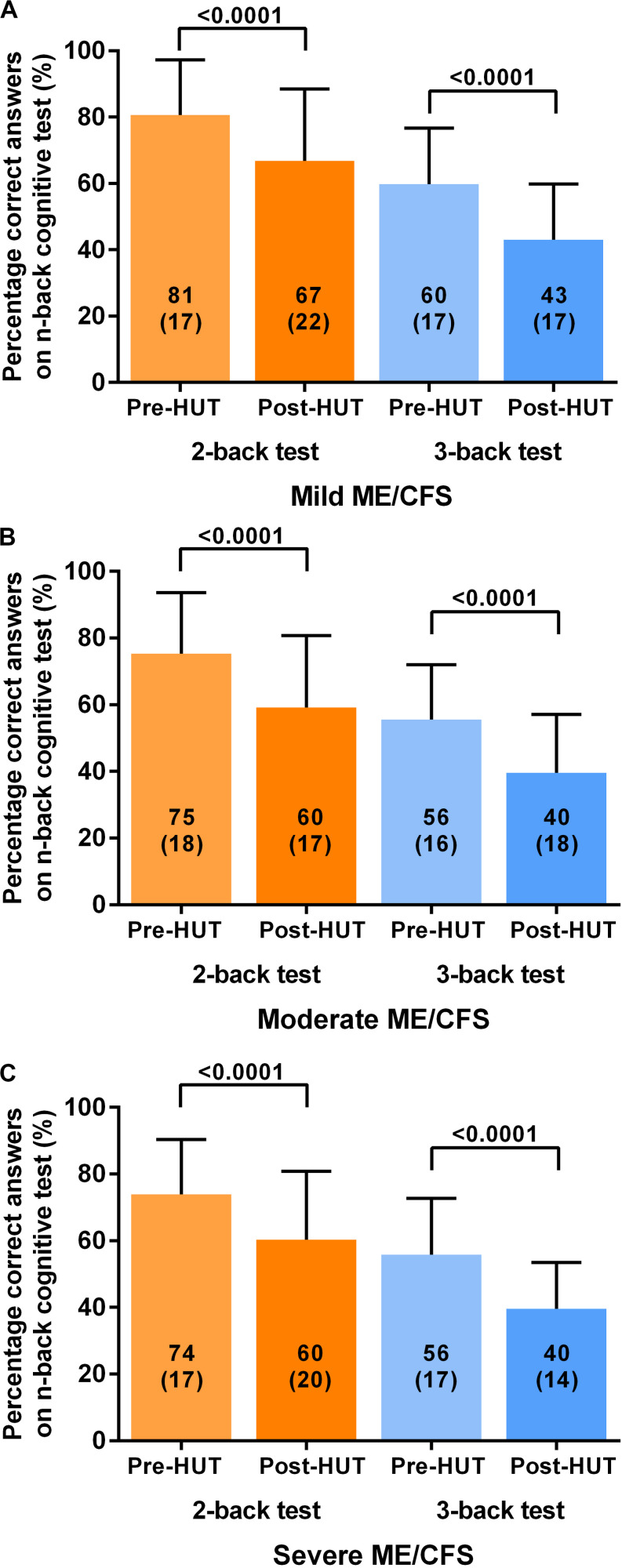
Shows the percentage of correct responses on 2-back and 3-back test before and after head-up-tilt testing, for the three disease severity grading of ME/CFS: patients with mild disease **(A)**, moderate disease **(B)**, and severe disease **(C)**. HUT: head-up tilt test.

**FIGURE 4:**
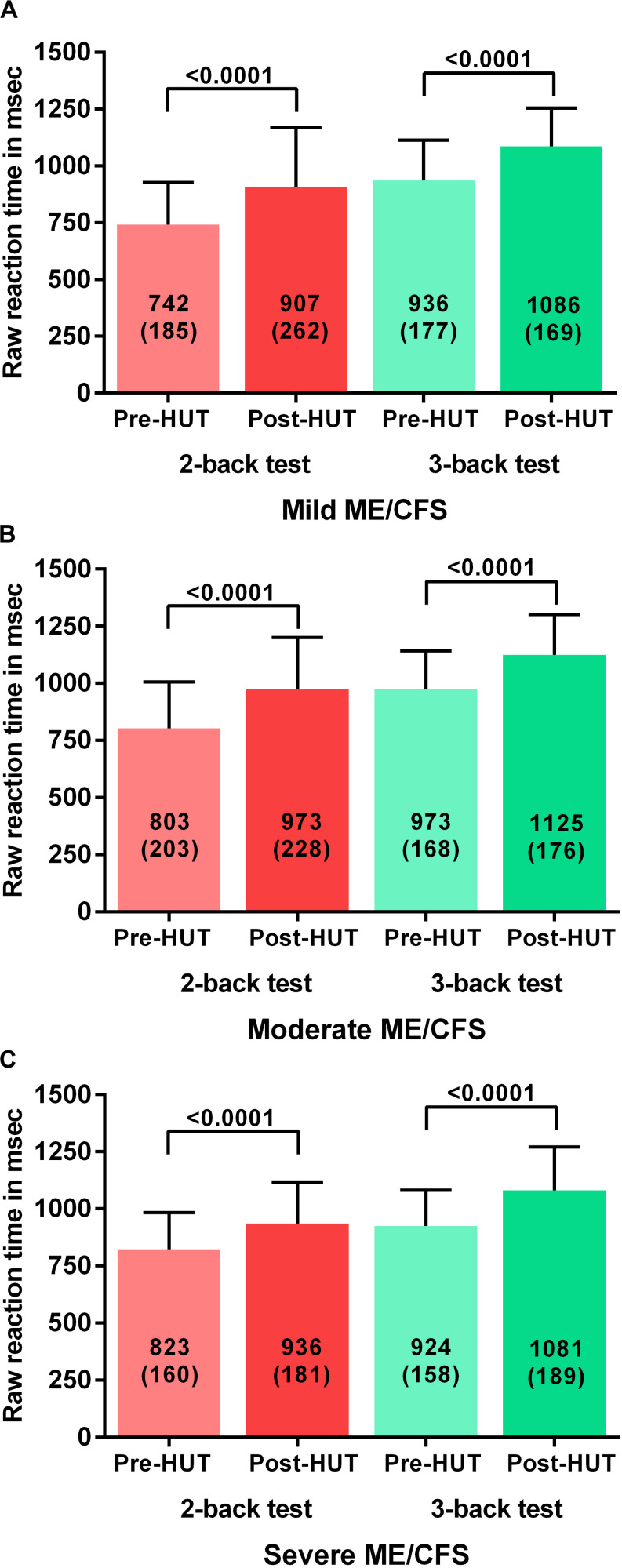
Shows the raw reaction time on 2-back an 3-back test before and after head-up-tilt testing, for the three disease severity grading of ME/CFS: patients with mild disease **(A)**, moderate disease **(B)**, and severe disease **(C)**. HUT: head-up tilt test.

Table 4 shows the N-back results of the patients with mild, moderate, and severe disease defined by the ME criteria. All N-back results (percent correct responses and raw reaction times for both 2-back and 3-back) were compared pre- and post-HUT. All parameters changed highly significantly different (*p* all <0.0001). The 2-way ANOVA showed no significant within group differences and no significant interaction effect between the three disease severity groups and the pre- and post-HUT N-back results for both 2-back and 3-back. No significant differences were found comparing the three groups. Figure 3 shows the percentage of correct responses of 2- and 3-back tests before and after HUT in ME/CFS patients with mild disease (Figure 3A), moderate disease (Figure 3B) and severe disease (Figure 3C). In all three groups a significant reduction of the percent correct responses was found (all *p* < 0.0001). Figure 4 shows the raw reaction time of 2- and 3-back tests before and after HUT in ME/CFS patients with mild disease (Figure 4A), moderate disease (Figure 4B) and severe disease (Figure 4C). In all three groups a significant reduction of the percent correct responses was found (all *p* < 0.0001). The 2-way ANOVA showed no significant within group differences and no significant interaction effect between the three hemodynamic profiles and the three severity groups. In patients with or without fibromyalgia no significant differences were found between the two groups (data not shown). In patients with or without SSRI’s no significant differences were found between the two groups (data not shown).

**TABLE 4:** N-back results pre- and post-HUT in ME/CFS patients with a mild, a moderate or a severe degree of ME/CFS.

**N-back results**	**Group 1 mild**	**Group 2 moderate**	**Group 3 severe**	**2-way ANOVA and *post hoc* Holm–Sidak test**
2 Back test	*n* = 49	*n* = 55	*n* = 24	
% Correct response pre-HUT	81 (17)	75 (18)	74 (17)	*F* (2, 250) = 0.018; *p* = 0.98.
% Correct response post-HUT	67 (22)	60 (17)	60 (20)	
Raw reaction time pre-HUT	742 (185)	803 (203)	823 (160)	*F* (2, 250) = 0.41; *p* = 0.66.
Raw reaction time post-HUT	907 (262)	973 (228)	936 (181)	
3 Back test				
% Correct response pre-HUT	60 (17)	56 (16)	56 (17)	*F* (2, 250) = 0.022; *p* = 0.98.
% Correct response post-HUT	43 (17)	40 (18)	40 (14)	
Raw reaction time pre-HUT	936 (177)	973 (168)	924 (158)	*F* (2, 250) = 0.0081; *p* = 0.99.
Raw reaction time post-HUT	1086 (169)	1125 (176)	1081 (189)	

*Data represent mean (SD). Raw reaction time is in m/s. HUT: head-up tilt test.*

## Discussion

The main finding of this study is that in adults with ME/CFS, orthostatic stress testing is followed by a deterioration of cognitive function as measured by a visual 2- and 3-back memory test. This deterioration of cognitive function was independent of the hemodynamic outcome of the HUT test. There was no difference in correct answers or raw reaction time between patients with no abnormalities in heart rate and blood pressure, POTS or orthostatic hypotension. A relation with the decline in cerebral blood flow during HUT – irrespective of heart rate and blood pressure changes – as consequence of the orthostatic stress may explain the deterioration in working memory. Compared to pre-HUT values, the number of correct answers diminished and the raw reaction time increased post-HUT. These findings are strengthened by the relatively large sample size compared to other studies of cognitive function in ME/CFS.

We elected to use the N-back test because Cockshell and Matthias showed in a meta-analysis that six out of the eight cognitive domains studied were significantly different in ME/CFS patients compared to healthy controls (Cockshell and Mathias, 2010). The N-back test assesses four of these six domains: reaction time, attention, memory, and motor functioning. Moreover, responses of the N-back test in healthy controls are correlated with activation in several brain regions as shown by neuro-imaging (Owen et al., 2005).

Several studies have used the N-back test in evaluating ME/CFS patients (Caseras et al., 2006; Stewart et al., 2012; Medow et al., 2014). The study of Caseras et al. (2006) investigated 17 ME/CFS patients and 12 healthy controls using a verbal N-back test administered while participants were supine. No differences between the ME/CFS patients and controls were found in the accuracy and the reaction times of the 2- and 3-back tests (Caseras et al., 2006). Nevertheless, trend analyses of task load, as detected by functional MRI imaging, demonstrated statistically significant differences in brain activation between the two groups with increasing task demands. Stewart et al. (2012) confirmed the absence of supine differences in the proportion of correct answers and reaction times between ME/CFS patients with co-morbid POTS and healthy controls. In contrast, with upright positioning using various degrees of tilting, Stewart et al., found a progressive worsening in the number of correct answers and an increase in reaction times in ME/CFS/POTS patients (*n* = 25) in contrast to controls (*n* = 20). They hypothesized that the cognitive impairment was caused by a reduction in cerebral blood flow as was demonstrated using transcranial Doppler flow velocities in ME/CFS/POTS patients and controls during HUT (Stewart et al., 2012).

The current study shows that the cognitive abnormalities in ME/CFS are not restricted to those with POTS. We have previously shown that ME/CFS patients with a normal HR and BP response to a 30 min HUT nonetheless have a significant reduction in cerebral blood flow (CBF) as measured by extracranial Doppler imaging of the internal carotid and vertebral arteries (van Campen et al., 2020). Healthy individuals develop a 7% CBF reduction, whereas those with ME/CFS develop a 28% reduction overall, 24% in those with normal HR/BP responses and 29% in those with POTS or orthostatic hypotension. Although the earlier study confirmed less of a decline in CBF in those with a normal HR and BP response to HUT, the current study identified no significant differences in 2-back and 3-back correct answers or reaction times between those with normal HR/BP and those with POTS. This suggests that there may be an important threshold of reduced CBF that is associated with the declines in cognitive performance.

Previous studies have described deterioration of the number of correct responses and an increase in reaction times in ME/CFS patients during HUT. Our study shows that similar findings are observed shortly after HUT (start of the N-back tests within 5 min), suggesting that the effects of orthostatic stress persist for a period of time following HUT. The IOM report mentions: “Clinicians who evaluate those with orthostatic intolerance recognize that individuals with ME/CFS can develop an exacerbation of their typical symptoms not just during the head-up tilt test but for several days afterward. The committee’s literature search did not identify any publications describing this observation more formally” [(IOM) 2015]. Previous studies have quantitatively demonstrated a decrease in cognitive functioning after physical exertion in ME/CFS patients (Blackwood et al., 1998; LaManca et al., 1998). Whether physical stress exerts the same cognitive dysfunction as orthostatic stress needs to be studied in the future.

Post exertional malaise (PEM) is an exacerbation of some or all of an individual’s ME/CFS symptoms that occurs after physical or cognitive exertion and leads to a reduction in functional ability (Carruthers et al., 2003). Some studies have shown that PEM may occur quickly, within 30 min of exertion (Blackwood et al., 1998) while others have found that patients may experience a worsening of symptoms 1–7 days after exertion (Sorensen et al., 2003; Yoshiuchi et al., 2007; van Oosterwijck et al., 2010; White et al., 2010). The results of our study suggest that post exertional malaise may start immediately after completion of the orthostatic stress test. Future studies will have to address whether the exacerbation in cognitive performance extends beyond 24 h.

Christodoulou et al. (1998) studied the relation between disease severity in ME/CFS and cognitive function using the California verbal learning test (CVLT). Patients reporting less activity were found to have worse test results than patients with relatively more activity. Using the 2- and 3-back test and grading severity according to the ME criteria, we could not replicate their findings (see Table 4 and Figures 3, 4; Carruthers et al., 2011). The differences between these studies might be explained by differences in tests, severity grading and sample size.

### Limitations

No comparison with healthy controls was available. However, in the study of the N-back test in healthy controls during HUT was not different from the supine N-back test Medow et al. (2014). Whether cognitive function results before and after HUT or before and after another stressor can be extrapolated to daily life remains also to be studied. In the present study patients performed the N-back tests while seated. There are suggestions that differences between ME/CFS patients and controls might be due to performing tests seated while no differences might be present in the supine position (Deluca et al., 1993; Deluca et al., 1995; Vollmer-Conna et al., 1997; Stewart et al., 2012). This hypothesis needs to be tested. We only tested ME/CFS patients with a clinical suspicion of OI who underwent HUT. The N-back tests pre- and post-HUT in ME/CFS patients without OI symptoms need to be evaluated. We did not evaluate the length of time that cognitive testing remains abnormal following HUT, but this deserves further study. We used an on-line, readily available version of the N-back test. We are not aware of a comparison of this test with other versions of the N-back test. Although different forms of the N-back test might have other stimulus exposure times, resulting in a different percentage of correct answers, in the present study patients were their own comparison, which validates the outcomes of a worse performance post-HUT.

## Conclusion

Using a visual 2- and 3-back test, this study shows that working memory is impaired shortly after orthostatic stress testing in ME/CFS patients, extending observations from previous small studies that working memory is impaired during orthostatic stress. Our results are consistent with the observation that PEM can start immediately after an orthostatic stress.

## Data Availability Statement

The raw data supporting the conclusions of this article will be made available by the authors, without undue reservation, to any qualified researcher.

## Ethics Statement

The studies involving human participants were reviewed and approved by ethics committee of the Slotervaart Hospital P1450. The patients/participants provided their written informed consent to participate in this study.

## Author Contributions

CC, PR, FWAV, and FCV conceived the study. CC and FCV collected the data. CC performed the primary data analysis. FCV, FWAV, and PR performed the secondary data analyses. All authors were involved in the drafting and review of the manuscript.

## Conflict of Interest

The authors declare that the research was conducted in the absence of any commercial or financial relationships that could be construed as a potential conflict of interest.

## References

[B1] Blackwood S. K. MacHale S. M. Power M. J. Goodwin G. M. Lawrie S. M. (1998). Effects of exercise on cognitive and motor function in chronic fatigue syndrome and depression. *J. Neurol. Neurosurg. Psychiatry* 65 541–546. 9771781 10.1136/jnnp.65.4.541 PMC2170292

[B2] Bou-Holaigah I. Rowe P. C. Kan J. Calkins H. (1995). The relationship between neurally mediated hypotension and the chronic fatigue syndrome. *JAMA* 274 961–967. 7674527

[B3] Briggs J. D. (1970). *1969 Activities of the WHO International Reference Center for Diagnosis of Vectors*. Switzerland: World Health Organization Available online at: https://apps.who/int/handle/10665/161266

[B4] Carruthers B. M. De Merileir K. L. Peterson D. L. Klimas N. G. Lerner A. M. Bested A. C. (2003). Myalgic encephalomyelitis/chronic fatigue syndrome: clinical working case definition, diagnostic and treatment protocols. *J. Chronic Fatigue Syndr.* 11 7–116.

[B5] Carruthers B. M. Klimas N. G. Mena I. Bell D. S. Lewis D. Light A. R. (2011). Myalgic encephalomyelitis: international consensus criteria. *J. Intern. Med.* 270 327–338. 10.1111/j.1365-2796.2011.02428.x 21777306 PMC3427890

[B6] Caseras X. David M. C. Giampietro V. Rimes K. A. Brammer M. Zelaya F. (2006). Probing the working memory system in chronic fatigue syndrome: a functional magnetic resonance imaging study using the n-back task. *Psychosom. Med.* 68 947–955. 17079703 10.1097/01.psy.0000242770.50979.5f

[B7] Christodoulou C. Deluca J. Lange G. Johnson S. K. Sisto S. A. Korn L. (1998). Relation between neuropsychological impairment and functional disability in patients with chronic fatigue syndrome. *J. Neurol. Neurosurg. Psychiatry* 64 431–434. 9576531 10.1136/jnnp.64.4.431 PMC2170049

[B8] Claypoole K. H. Noonan C. Mahurin R. K. Goldberg J. Erickson T. Buchwald D. (2007). A twin study of cognitive function in chronic fatigue syndrome: the effects of sudden illness onset. *Neuropsychology* 21 507–513. 17605583 10.1037/0894-4105.21.4.507

[B9] Cockshell S. J. Mathias J. L. (2010). Cognitive functioning in chronic fatigue syndrome: a meta-analysis. *Psychol. Med.* 40 1253–1267. 20047703 10.1017/S0033291709992054

[B10] Constant E. L. Adam S. Gillain B. Lambert M. Masquelier E. Seron X. (2011). Cognitive deficits in patients with chronic fatigue syndrome compared to those with major depressive disorder and healthy controls. *Clin. Neurol. Neurosurg.* 113 295–302. 21255911 10.1016/j.clineuro.2010.12.002

[B11] De Lorenzo F. Hargreaves J. Kakkar V. V. (1997). Pathogenesis and management of delayed orthostatic hypotension in patients with chronic fatigue syndrome. *Clin. Auton. Res.* 7 185–190. 9292244 10.1007/BF02267980

[B12] Deluca J. Johnson S. K. Beldowicz D. Natelson B. H. (1995). Neuropsychological impairments in chronic fatigue syndrome, multiple sclerosis, and depression. *J. Neurol. Neurosurg Psychiatry* 58 38–43. 7823065 10.1136/jnnp.58.1.38 PMC1073266

[B13] Deluca J. Johnson S. K. Natelson B. H. (1993). Information processing efficiency in chronic fatigue syndrome and multiple sclerosis. *Arch. Neurol.* 50 301–304. 8442710 10.1001/archneur.1993.00540030065016

[B14] Eeftinck S. D. W. van Lieshout J. J. van den Meiracker A. H. Wesseling K. R. Blanc S. Wieling W. (2009). Nexfin noninvasive continuous blood pressure validated against Riva-Rocci/Korotkoff. *Am. J. Hypertens.* 22 378–383. 10.1038/ajh.2008.368 19180062

[B15] FDA (2013). *The Voice of the Patient: Chronic Fatigue Syndrome and Myalgic Encepalomyelitis.* Bethesda, MD: FDA.

[B16] Freeman R. Komaroff A. L. (1997). Does the chronic fatigue syndrome involve the autonomic nervous system? *Am. J. Med.* 102 357–364. 9217617 10.1016/s0002-9343(97)00087-9

[B17] Freeman R. Wieling W. Axelrod F. B. Benditt D. G. Benarroch E. Biaggioni I. (2011). Consensus statement on the definition of orthostatic hypotension, neurally mediated syncope and the postural tachycardia syndrome. *Auton. Neurosci.* 161 46–48. 10.1016/j.autneu.2011.02.004 21393070

[B18] Fukuda K. Straus S. E. Hickie I. Sharpe M. C. Dobbins J. G. Komaroff A. (1994). The chronic fatigue syndrome: a comprehensive approach to its definition and study. International chronic fatigue syndrome study group. *Ann. Intern. Med.* 121 953–959. 7978722 10.7326/0003-4819-121-12-199412150-00009

[B19] Institute of Medicine [IOM], (ed.). (2015). *Beyond Mayalgic Encephalomyelitis/Chronic Fatigue Syndrome: Redefining an Illness.* Washington DC: The National Academies Press, 10.17226/19012 25695122

[B20] LaManca J. J. Sisto S. A. Deluca J. Johnson S. K. Lange G. Pareja J. (1998). Influence of exhaustive treadmill exercise on cognitive functioning in chronic fatigue syndrome. *Am. J. Med.* 105 59S–65S. 9790484 10.1016/s0002-9343(98)00171-5

[B21] Larun L. Malterud K. (2007). Identity and coping experiences in Chronic Fatigue Syndrome: a synthesis of qualitative studies. *Patient. Educ. Couns.* 69 20–28. 17698311 10.1016/j.pec.2007.06.008

[B22] Low P. A. Sandroni P. Joyner M. Shen W. K. (2009). Postural tachycardia syndrome (POTS). *J. Cardiovasc. Electrophysiol.* 20 352–358. 10.1111/j.1540-8167.2008.01407.x 19207771 PMC3904426

[B23] Mahurin R. K. Claypoole K. H. Goldberg J. H. Arguelles L. Ashton S. Buchwald D. (2004). Cognitive processing in monozygotic twins discordant for chronic fatigue syndrome. *Neuropsychology* 18 232–239. 15099145 10.1037/0894-4105.18.2.232

[B24] Martina J. R. Westerhof B. E. van Goudoever J. de Beaumont E. M. Truijen J. Kim Y. S. (2012). Noninvasive continuous arterial blood pressure monitoring with Nexfin(R). *Anesthesiology* 116 1092–1103. 10.1097/ALN.0b013e31824f94ed 22415387

[B25] Medow M. S. Sood S. Messer Z. Dzogbeta S. Terilli C. Stewart J. M. (2014). Phenylephrine alteration of cerebral blood flow during orthostasis: effect on n-back performance in chronic fatigue syndrome. *J. Appl. Physiol.* 117 1157–1164. 10.1152/japplphysiol.00527.2014 25277740 PMC4233252

[B26] Owen A. M. McMillan K. M. Laird A. R. Bullmore E. (2005). N-back working memory paradigm: a meta-analysis of normative functional neuroimaging studies. *Hum. Brain Mapp.* 25 46–59. 10.1002/hbm.20131 15846822 PMC6871745

[B27] Sheldon R. S. Blair P. G. Carlos A. M. Julian M. S. Dennis H. L. Karen J. F. (2015). 2015 heart rhythm society expert consensus statement on the diagnosis and treatment of postural tachycardia syndrome, inappropriate sinus tachycardia, and vasovagal syncope. *Heart Rhythm.* 12 e41–e63. 10.1016/j.hrthm.2015.03.029 25980576 PMC5267948

[B28] Sorensen B. Streib J. E. Strand M. Make B. Giclas P. C. Fleshner M. (2003). Complement activation in a model of chronic fatigue syndrome. *J. Allergy Clin. Immunol.* 112 397–403. 12897748 10.1067/mai.2003.1615

[B29] Stewart J. M. Gewitz M. H. Weldon A. Arlievsky N. Li K. Munoz J. (1999). Orthostatic intolerance in adolescent chronic fatigue syndrome. *Pediatrics* 103 116–121. 9917448 10.1542/peds.103.1.116

[B30] Stewart J. M. Medow M. S. Messer Z. R. Baugham I. L. Terilli C. Ocon A. J. (2012). Postural neurocognitive and neuronal activated cerebral blood flow deficits in young chronic fatigue syndrome patients with postural tachycardia syndrome. *Am. J. Physiol. Heart Circ. Physiol.* 302 H1185–H1194. 10.1152/ajpheart.00994.2011 22180650 PMC3311460

[B31] Streeten D. H. Bell D. S. (1999). Long- and short-term blood pressure and RR-interval variability and psychosomatic distress in chronic fatigue syndrome. *Clin. Sci.* 97 319–322. 10576962 10.1042/cs19990067

[B32] Togo F. Lange G. Natelson B. H. Quigley K. S. (2015). Attention network test: assessment of cognitive function in chronic fatigue syndrome. *J. Neuropsychol.* 9 1–9. 10.1111/jnp.12030 24112872 PMC4159443

[B33] van Campen C. L. M. C. Verheugt F. W. A. Rowe P. C. Visser F. C. (2020). Cerebral blood flow is reduced in ME/CFS during head-up tilt testing even in the absence of hypotension or tachycardia: a quantitative, controlled study using doppler echography. *Clin. Neurophysiol. Pract.* 5 50–58. 10.1016/j.cnp.2020.01.003 32140630 PMC7044650

[B34] van Campen C. L. M. C. Verheugt F. W. A. Visser F. C. (2018). Cerebral blood flow changes during tilt table testing in healthy volunteers, as assessed by doppler imaging of the carotid and vertebral arteries. *Clin. Neurophysiol. Pract.* 3 91–95. 10.1016/j.cnp.2018.02.004 30215015 PMC6133915

[B35] van Oosterwijck J. Nijs J. Meeus M. Lefever I. Huybrechts L. Lambrecht L. (2010). Pain inhibition and postexertional malaise in myalgic encephalomyelitis/chronic fatigue syndrome: an experimental study. *J. Intern. Med.* 268 265–278. 20412374 10.1111/j.1365-2796.2010.02228.x

[B36] Vollmer-Conna U. Wakefield D. Lloyd A. Hickie I. Lemon J. Bird K. D. (1997). Cognitive deficits in patients suffering from chronic fatigue syndrome, acute infective illness or depression. *Br. J. Psychiatry* 171 377–381. 9373430 10.1192/bjp.171.4.377

[B37] White A. T. Light A. R. Hughen R. W. Bateman L. Martins T. B. Hill H. R. (2010). Severity of symptom flare after moderate exercise is linked to cytokine activity in chronic fatigue syndrome. *Psychophysiology* 47 615–624. 20230500 10.1111/j.1469-8986.2010.00978.x PMC4378647

[B38] Yoshiuchi K. Cook D. B. Ohashi K. Kumano H. Kuboki T. Yamamoto Y. (2007). A real-time assessment of the effect of exercise in chronic fatigue syndrome. *Physiol. Behav.* 92 963–968. 17655887 10.1016/j.physbeh.2007.07.001 PMC2170105

